# Comprehensive Evaluation of Cerebral Hemodynamics and Oxygen Metabolism in Revascularization of Asymptomatic High-Grade Carotid Stenosis

**DOI:** 10.1007/s00062-021-01077-3

**Published:** 2021-09-06

**Authors:** Bernardo Crespo Pimentel, Jan Sedlacik, Julian Schröder, Marlene Heinze, Leif Østergaard, Jens Fiehler, Christian Gerloff, Götz Thomalla, Bastian Cheng

**Affiliations:** 1 Department of Neurology, University Medical Centre Hamburg-Eppendorf, Hamburg, Germany; 2 Centre for the Developing Brain & Biomedical Engineering Department, School of Biomedical Engineering & Imaging Sciences, King’s College London, London, UK; 3 Center of Functionally Integrative Neuroscience and MINDLab, Institute of Clinical Medicine, Aarhus University, Aarhus, Denmark; 4 Department of Neuroradiology, Aarhus University Hospital, Aarhus, Denmark; 5 Department of Neuroradiology, University Medical Centre Hamburg-Eppendorf, Hamburg, Germany

**Keywords:** Capillary transit time heterogeneity, Atherosclerosis, Carotid artery stenting, Oxygen extraction fraction, Perfusion-weighted imaging

## Abstract

**Introduction:**

Revascularization procedures in carotid artery stenosis have shown a positive effect in the restoration of cerebral oxygen metabolism as assessed by T2’ (T2 prime) imaging as well as capillary homeostasis by measurement of capillary transit time heterogeneity (CTH); however, data in patients with asymptomatic carotid stenosis without manifest brain lesions are scarce.

**Patients and Methods:**

The effect of revascularization on the hemodynamic profile and capillary homeostasis was evaluated in 13 patients with asymptomatic high-grade carotid stenosis without ischemic brain lesions using dynamic susceptibility contrast perfusion imaging and oxygenation-sensitive T2’ mapping before and 6–8 weeks after revascularization by endarterectomy or stenting. The cognitive performance at both timepoints was further assessed.

**Results:**

Perfusion impairment at baseline was accompanied by an increased CTH (*p* = 0.008) in areas with a time to peak delay ≥ 2 s in the affected hemisphere compared to contralateral regions. Carotid intervention improved the overall moderate hemodynamic impairment at baseline by leading to an increase in normalized cerebral blood flow (*p* = 0.017) and a decrease in mean transit time (*p* = 0.027), oxygen extraction capacity (OEC) (*p* = 0.033) and CTH (*p* = 0.048). The T2’ values remained unchanged.

**Conclusion:**

This study presents novel evidence of a state of altered microvascular function in patients with high-grade carotid artery stenosis in the absence of ischemic brain lesions, which shows sustained normalization after revascularization procedures.

**Supplementary Information:**

The online version of this article (10.1007/s00062-021-01077-3) contains supplementary material, which is available to authorized users.

## Introduction

Large-vessel atherosclerotic disease is the leading cause of ischemic stroke worldwide [1]. Specifically high-grade asymptomatic internal carotid artery stenosis (ICAS) has an estimated prevalence of up to 3.1% in the general population and carries an annual stroke risk of approximately 2–5% [2, 3]. Although hemodynamic impairment and changes in oxygen metabolism play an important role in the risk of subsequent stroke in patients with symptomatic ICAS [4, 5], data from patients with asymptomatic disease are scarce and conflicting. Perfusion abnormalities, microvascular impairment and altered network integrity appear in these patients [6–10] and seem to play a role in the emergence of long-term comorbidities, such as white matter hyperintensities of presumed vascular origin and cognitive dysfunction [8–10]. Therefore, the term asymptomatic may carry a misleading connotation given the subtle, yet reproducible, pathophysiological changes in subjects with high-grade stenosis and absence of clinical stroke symptoms or lesions detected by imaging.

Magnetic resonance imaging (MRI) is increasingly used to characterize metabolic alterations related to a disturbed brain oxygen metabolism in chronic hypoperfusion. Quantitative T2 prime (qT2’) MRI uses the blood oxygen level-dependent effect to detect differences in the concentration of deoxygenated hemoglobin and might be considered a surrogate marker of oxygen extraction fraction (OEF) [11]. Previous studies showed an association between increased estimated OEF as measured by T2’ mapping and perfusion changes in a cohort of symptomatic and asymptomatic patients in the ipsilateral hemisphere to high-grade ICAS [12]. The T2’ values normalized during the first days after carotid intervention, affirming the role of revascularization treatment in restoring cerebral oxygen metabolism [13].

Both the heterogeneity of microvascular flow and the efficiency of oxygen extraction can be inferred by the retention of intravascular contrast media in dynamic susceptibility contrast (DSC) perfusion-weighted imaging [14]. It has been shown that capillary flow patterns affect the extraction of oxygen from blood at a given cerebral blood flow (CBF) and ultimately impact the cerebral metabolic rate of oxygen (CMRO_2_) [15]. The capillary transit time heterogeneity (CTH) is therefore a measure of the microvascular flow of erythrocytes and can be applied as a marker of capillary dysfunction [16]. Altered microvascular flow patterns are represented by an elevated CTH and have been demonstrated in several neurological conditions, such as stroke [16], dementia [17], and white matter disease [20]. In high-grade ICAS, CTH has shown to be reversible after revascularization and to be superior to Tmax in predicting functional outcome [18, 19].

In this study, a comprehensive evaluation of hemodynamics, oxygen metabolism and microvascular function in patients with asymptomatic unilateral high-grade ICAS before and after revascularization using qT2’ mapping and DSC perfusion-weighted imaging was aimed. The clinical significance of these findings was investigated through the assessment of cognitive performance. The hypothesis under test is that carotid revascularization leads to a homogenization of capillary transit times as well as a normalization of T2’ values, the latter reflecting the restoration of cerebral autoregulatory capacity.

## Patients and Methods

### Patients

Patients were prospectively recruited between August 2014 and October 2016 at the University Medical Center Hamburg-Eppendorf. Patients had Doppler/ultrasound evidence of unilateral high-grade ICAS (≥ 70% according to NASCET criteria) [20] and underwent revascularization treatment either by stenting or endarterectomy. Only asymptomatic patients with absent ischemic brain lesions on DWI and mild to moderate degree of white matter lesions in fluid attenuation inversion recovery (FLAIR) sequences were included. Patients with contralateral low-grade stenosis (< 50% according to NASCET) were also included. Study subjects were identified by incidental findings and excluded if they had history of stroke, dementia, depression, significant neurological disability, as well as any contraindications for a MRI scan. No iatrogenic ischemic lesions were detected on follow-up. Data from a subset of these patients have recently been reported by Schröder et al., demonstrating a significant hypoperfusion that reversed after revascularization in the middle cerebral artery (MCA) territory of the affected side [21]. The local ethics committee (Ethikkomission der Ärztekammer Hamburg) approved the study protocol. Informed consent was signed by every participant according to the Declaration of Helsinki.

### Magnetic Resonance Imaging

Patients underwent structural and perfusion MRI within 10 days before (first time point: TP1) and 6–8 weeks after revascularization procedures (second time point: TP2). All MRI scans were acquired with a 3T Siemens Scanner (Skyra, Siemens, Erlangen, Germany).

The MRI protocol included T1 MPRage (flip angle = 9°, TR = 2500 ms, TE = 2.12 ms, slice thickness = 0.9 mm, inversion time 1100 ms, matrix = 232 × 288, field of view, FOV = 193 × 293 mm^2^, acquisition time: 05:42 min), FLAIR (flip angle = 150°, TR = 9000 ms, TE = 90 ms, slice thickness = 5 mm, inversion time = 2500 ms, matrix = 320 × 270, FOV = 194 × 230 mm^2^, acquisition time: 02:08 min) and DSC perfusion-weighted imaging (flip angle = 90°,TR = 1920 ms, TE = 30 ms, slice thickness = 4 mm, matrix = 128 × 128, FOV = 240 × 240 mm^2^, acquisition time: 01:43 min). During dynamic acquisition, a single dose of 0.1 mmol/kg of a macrocyclic gadolinium contrast agent (DOTAREM®, Guerbet, Cincinnati, OH, USA) with a concentration of 0.5 mmol/mL was injected with a flow rate of 5 mL/s using an automatic injection pump.

The values of R2 and R2*, the inverse of transverse relaxation times T2 and T2* (or T2 “star”), respectively, were assessed by multi-echo TSE (echo times 12, 86, and 160 ms, TR = 6580 ms, matrix = 128 × 128, FOV = 240 × 240 mm^2^, slice thickness = 4 mm, acquisition time: 01:32 min) and multi-echo GRE (echo times 3, 8, 13, 18 and 23 ms, TR = 724 ms, matrix size = 128 × 128, FoV = 240 × 240 mm^2^; slice thickness = 4 mm; monopolar echo readout, acquisition time: 01:41 min) sequences. Magnitude and corresponding phase images were acquired to allow correction of field inhomogeneities in T2*.

### Image Processing

Perfusion series were motion-corrected prior to analysis and subsequently processed through the automated perfusion analysis software Cercare Medical (https://cercare-medical.com/). This software tool follows a parametric modelling approach for estimating CBF, CTH and cerebral blood volume (CBV) from DSC-MRI raw data [14, 22], and, derived from these, the oxygen extraction capacity (OEC), defined as the maximum OEF for a given CBF, as well as the maximum CMRO_2_ [15]. The model is composed of three parts: i) a model of oxygen extraction along a single capillary *Q* as function of transit time *t*, ii) a model of the capillary transit time distribution *h* (*t*), and iii) the resulting OEC defined as the sum of the single capillary contributions weighted by the capillary transit time distribution [23]. The Q (*t*) is modelled as a three-compartment model consisting of hemoglobin, tissue and blood plasma. The model assumes normal tissue oxygen tension, P_t_O_2_ = 25 mm Hg. The upper limit of the cerebral metabolic rate of oxygen that can be supported for P_t_O_2_ = 25 mm Hg is calculated as $\mathrm{CMR}O_{2}=Ca\cdot CBF\cdot OEC$, where *Ca* represents the arterial oxygen concentration. Arterial input functions were semi-automatically determined in the MCA territory contralateral to the stenotic side. All images were co-registered intraindividually to the FLAIR images.

For the generation of qT2’ maps, multi-echo turbo spin echo (TSE) and gradient echo (GRE) magnitude images of the first echo time were skull-stripped using the Brain Extraction Tool of the FMRIB Software Library [24] and transverse relaxation maps were calculated for the extracted brain data. The R2 and R2* maps were calculated voxel-wise by a previously described algorithm [25], which includes the removal of macroscopic field gradients and monoexponential fitting. To ensure congruency between R2 and R2* maps, skull-stripped multi-echo TSE and GRE magnitude images of the first echo time were rigidly registered using FMRIB’s Linear Image Registration Tool [26, 27]. The R2* map was then realigned with the R2 map using the transformation matrix obtained from the registration of the multi-echo TSE and GRE magnitude images and R2’ maps were calculated by the relation R2’ = R2* − R2. In order to be in line with the existing literature, we converted the relaxation rate maps into relaxation time maps using the following relation: *T2*’ $=\frac{1}{R2\text{'}}$. In addition, in order to minimize contribution of non-plausible T2’ values (e.g. iron accumulation in the basal ganglia) and avoid partial volume artifacts due to cerebrospinal fluid, qT2’ maps were thresholded through visual inspection at ≥ 30 ms and ≤ 225 ms.

**Fig. 1:**
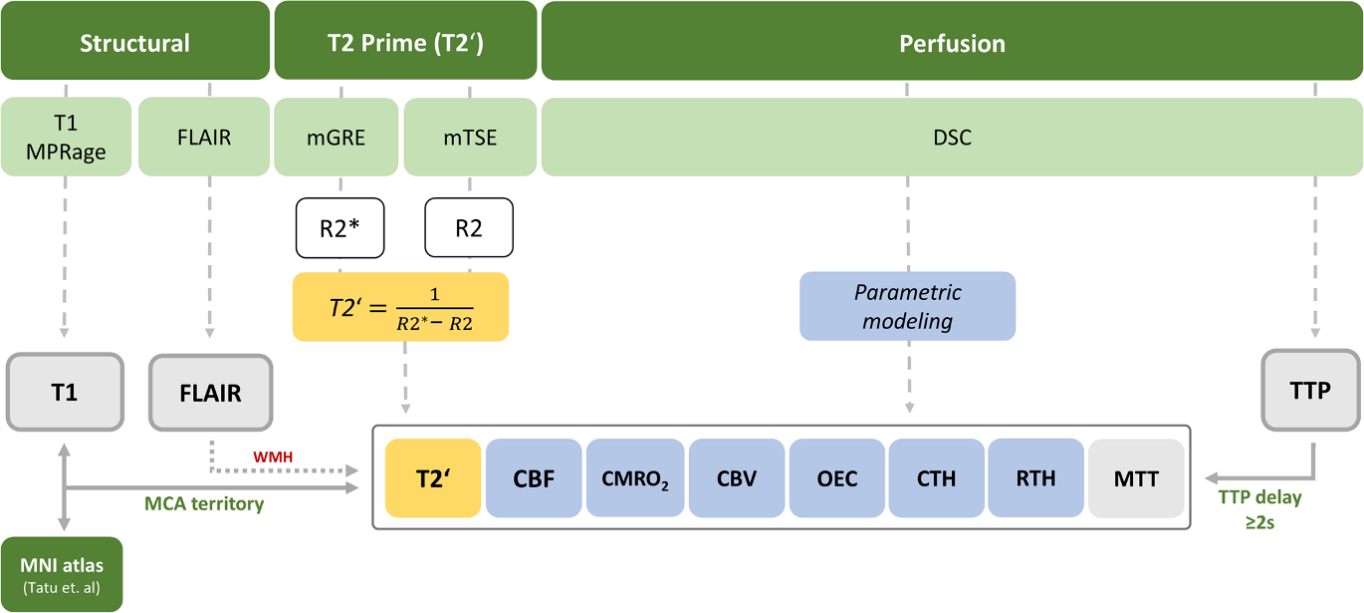
Overview of MRI protocol and analyzed parameters. Structural imaging including FLAIR and T1 MPRage allowed exclusion of ischemic lesions and generation of white matter hyperintensity (WMH) masks. By parametric modeling of raw dynamic susceptibility contrast (DSC) MRI data, cerebral blood flow (CBF), cerebral metabolic rate of oxygen (CMRO_2_), cerebral blood volume (CBV), oxygen extraction capacity (OEC), capillary transit time heterogeneity (CTH), mean transit time (MTT) and relative transit time heterogeneity (RTH) maps were calculated. For the generation of T2 prime (T2’) maps, multi-echo turbo spin echo (mTSE) and gradient echo (mGRE) sequences were employed with the intermediate generation of R2 and R2* maps. Time to peak (TTP) was used to generate individual areas with relative hypoperfusion ipsilateral in the stenotic side (using a TTP threshold of 2s in relation to the mean value of a standardized ROI on the contralateral side) and a previously published MNI atlas was employed to generated individual masks of the median cerebral artery (MCA) vascular territory. The MCA territory and perfusion lesion masks were applied to each of the eight hemodynamic maps for all participants while WMH were masked out

The FLAIR images were thoroughly inspected for T2-hyperintense lesions in both hemispheres and these lesions were subsequently automatically segmented using the Brain Intensity AbNormality Classification algorithm of the FMRIB software [28] and masked out from the analyzed maps. We first analyzed the variation of absolute values between hemispheres at baseline and longitudinally across time points. Due to the semiquantitative nature of CBF and CBV and the dependence of CMRO_2_ on CBF, we normalized studied parameters for longitudinal analysis (termed nqT2’, nCBF, nCBV, nMTT, nTTP, nOEC, nCMRO_2_, nCTH, and nRTH) by calculating interhemispheric ratios (mean values from regions of interest, ROI) ipsilateral to stenosis divided by values from the contralateral corresponding regions (Fig. 1).

### Region of Interest Definition

Two major analyses were performed. In the first analysis, the analyzed parameters were assessed in the MCA territory in both affected and unaffected hemispheres, irrespective of perfusion abnormalities. The MCA territory mask was created in Montreal Neurological Institute (MNI) space based on an available atlas [29]. Masks were registered to the individual space of each time point using T1 as an intermediate image, as well as to the qT2’ maps through co-registration with the TSE first echo time.

**Fig. 2:**
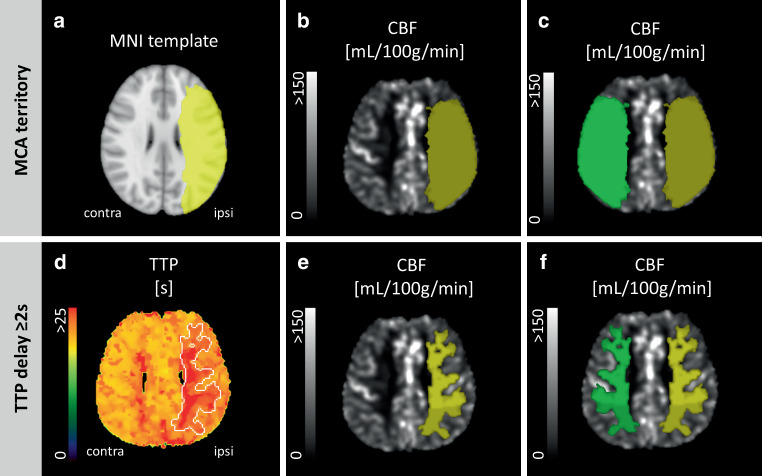
In the first analysis, a pre-established middle cerebral artery (MCA) territory mask in MNI space (**a**) was transferred to the stenotic side of all hemodynamic maps (ipsi). Cerebral blood flow (CBF) is representatively depicted in this figure, where the MCA mask (**b**, in *yellow*) was mirrored to the contralateral hemisphere (**c**, in *green*). In the second analysis, areas with a time-to-peak (TTP) delay ≥ 2 s in the stenotic side (in relation to the standardized mean region of interest (ROI) value on the contralateral hemisphere) were manually delineated in 3–5 adjacent slides in the TTP map (**d**), creating a mask that was subsequently transferred to the ipsilateral side of the hemodynamic maps (**e**, in *yellow*) and finally mirrored to the contralateral side (**f**, in *green*). Ipsi indicates regions of interest in the hemisphere ipsilateral to the stenosis, Contra refers to the regions of interest in the hemisphere contralateral to the stenosis

In the second analysis, areas of baseline perfusion delay outlined in the TTP map were used as ROIs to measure the parameters of interest in the different time points. Baseline TTP maps were assessed by placing a standardized circular ROI in the contralateral MCA territory. The images were then thresholded according to the mean ROI value (assuming that it indicates normal TTP values). The ≥ 2 s threshold (in relation to the standardized mean ROI value) ensured most significant changes in qT2’ values in previous studies [12, 13] and provided well defined ROIs in all our subjects. Regions with TTP delay ≥ 2 s were manually delineated in 3–5 adjacent slices and transferred to the co-registered maps in both time points using FLAIR as an intermediate image. The ROI delineation is outlined in Fig. 2.

### Cognitive Assessment

A battery of neuropsychological tests was performed in all participants within 10 days before and 6–10 weeks after carotid revascularization by a neurologist trained and experienced in testing cognitive performance. The mini mental state examination (MMSE) and the dementia detection test (DemTect) were employed to evaluate global cognition. In addition, executive function was assessed with the trail-making test A/B and Stroop test. The final score of MMSE and DemTec and the time values required to complete the trail-making and Stroop tests were used for analysis.

### Statistical Analysis

Statistical analysis was performed using R Studio (R software package, v. 3.6.2; R Foundation for Statistical Computing, Vienna, Austria) [30]. Due to the non-normal distribution of baseline absolute parameter values, interhemispheric and longitudinal comparisons were performed with the paired Wilcox-Mann-Whitney test. Normalized parameter values were normally distributed according to visual assessment with quantile to quantile plots and therefore a paired *t*-test was applied for timepoint comparison. Pearson’s coefficient was used for testing correlations and Bonferroni correction was applied to adjust for multiple testing. All tests were 2‑sided and statistical significance was set to an alpha of 0.05.

## Results

Overall, 30 patients were enrolled in this study. Only 15 patients underwent DSC perfusion-weighted imaging at both time points and from these, 2 patients were excluded due to insufficient image quality. Thus, a total of 13 patients with complete follow-up were included in the DSC perfusion analysis. Of these, one patient lacked T2’ imaging, making up a total of 12 patients for the T2’ analysis.

Median patient age was 69 years (IQR 62–72 years), with a range of 52–87 years and 85% of patients were male (*n* = 11). Only 3 patients (23.08%) presented contralateral low-grade ICA stenosis. Patient demographic characteristics and medical history are displayed in Table 1.

**Table 1:** Demographic and clinical data

	Baseline	After 6–8 weeks	*p*
*Age (median, IQR)*	69.0 (62.0–72.0) years	–	–
*Sex (n)*	2 women, 11 men	–	–
*Vascular risk factors (n, %)*	–	–	–
Hypertension	11 (84.6)	–	–
Diabetes mellitus	4 (30.8)	–	–
Dyslipidemia	6 (46.2)	–	–
Smoking	3 (23.1)	–	–
*Degree of stenosis (median, IQR)*	70.0 (70.0–80.0) %	–	–
*Therapy*	10 CAE, 3 CAS	–	–
*Education (median, range)*	14.3 (12.8–18.0) years	–	–
*Cognitive testing*	–	–	–
MMSE (median, IQR)	27.0 (26.0–28.0)	27.5 (26.0–28.3)	0.959
DemTect (median, IQR)	15.0 (13.0–16.0)	14 (13.0–18.0)	1.000
TMT‑A (mean ± SD)	42.2 ± 11.4	38.76 ± 13.9	0.252
TMT‑B (mean ± SD)	120.6 ± 63.5	110.5 ± 72.2	0.103
FWT‑I (mean ± SD)	16.53 ± 5.3	16.8 ± 3.6	0.180
FWT-II (mean ± SD)	25.1 ± 5.8	25.5 ± 7.4	0.719
FWT-III (mean ± SD)	52.6 ± 16.0	49.6 ± 14.2	0.890

*CAE* carotid artery endarterectomy, *CAS* carotid artery stenting, *MMSE* mini mental state examination, *TMT* trail-making test, *FWT* Faber-Wort test, *DemTect* Dementia Detection Test, *IQR* interquartile range, *SD* standard deviation

### Perfusion Analysis

At baseline, there was no significant difference in any of the measured parameters between the stenosed and contralateral hemispheres in the whole MCA territory (see Table 2). After carotid intervention, no significant changes in the non-normalized parameters were seen between timepoints (see Supplemental Table 1). After normalization of the analyzed parameters, there was a significant reduction of 4.78% in nMTT (*p* = 0.007) and of 1.93% in nOEC (*p* = 0.007), while the other variables remained unchanged (see Supplemental Table 3).

**Table 2:** Hemisphere comparison of non-normalized parameters in the MCA territory

	TP1 After 6–8 weeks			TP2		
	*Ipsi*	*Contra*	*p*	*Ipsi*	*Contra*	*p*
CBF _(mL/100 g per min)_	97.28 (64.05)	97.38 (97.70)	1.000	76.29 (82.47)	75.93 (85.62)	0.685
CBV _(mL/100g)_	177.42 (228.29)	173.71 (224.22)	0.687	164.54 (133.12)	162.29 (137.58)	0.109
MTT _(s)_	3.05 (3.19)	2.76 (2.52)	0.264	3.45 (3.67)	47.59 (26.46)	0.454
OEC _(%)_	44.59 (18.23)	42.46 (14.61)	0.287	46.94 (15.87)	47.59 (16.74)	0.735
CTH _(s)_	3.41 (4.37)	3.05 (3.91)	0.310	3.85 (5.89)	3.87 (5.65)	0.094
CMRO_2 (mL/100_ _mL per min)_	4.25 (3.26)	4.24 (3.15)	0.880	3.97 (3.01)	4.04 (3.20)	0.305
T2’ _(ms)_	112.78 (25.92)	111.84 (30.22)	0.977	109.35 (20.67)	109.85 (26.46)	0.569
RTH	1.13 (0.26)	1.13 (0.30)	0.724	1.16 (0.45)	1.18 (0.42)	0.216

*CBF* cerebral blood flow, *CBV* cerebral blood volume, *MTT* median transit time, *OEC* oxygen extraction capacity, *CTH* capillary transit time heterogeneity, *CMRO*_*2*_ cerebral metabolic rate of oxygen, *T2’* T2 prime, *RTH* relative transit time heterogeneity

Non-normalized values are presented as median (interquartile range). *Ipsi* indicates regions of interest in the hemisphere ipsilateral to the stenosis, *Contra* refers to the regions of interest in the hemisphere contralateral to the stenosis

**Fig. 3:**
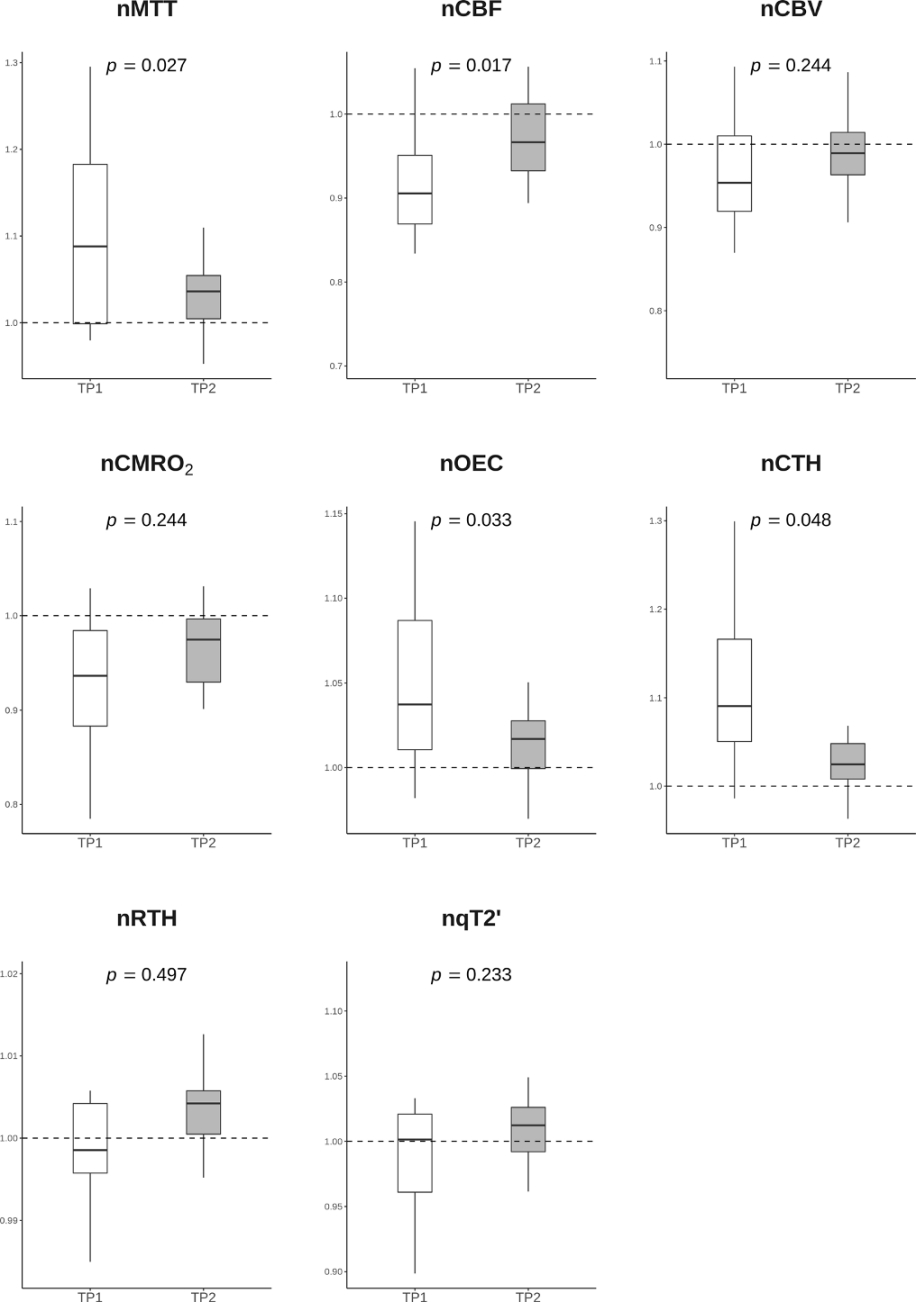
Boxplots for normalized hemodynamic and oxygen-metabolic parameters. The values obtained from areas with a time-to-peak (TTP) delay ≥ 2 s before (TP1) and 6–8 weeks after carotid revascularization (TP2). The Y‑axes represent the relative values with respect to the contralateral hemisphere. *nMTT* median transit time, *nCBF* cerebral blood flow, *nCBV* cerebral blood volume, *nCMRO*_*2*_ cerebral metabolic rate of oxygen, *nOEC* oxygen extraction capacity, *nCTH* capillary transit time heterogeneity, *nRTH* relative transit time heterogeneity, *T2’* T2 prime

**Fig. 4:**
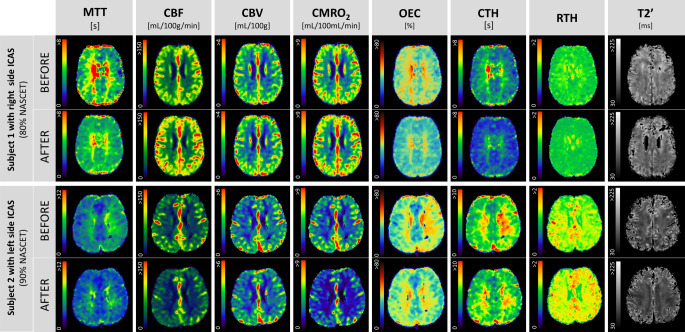
Illustration of hemodynamic and oxygen-metabolic color maps from two subjects with internal carotid artery stenosis (ICAS) before and after carotid revascularization. Color bars located left to the maps represent the range of voxel values. *MTT* median transit time, *CBF* cerebral blood flow, *CBV* cerebral blood volume, *CMRO*_*2*_ cerebral metabolic rate of oxygen, *OEC* oxygen extraction capacity, *CTH* capillary transit time heterogeneity, *RTH* relative transit time heterogeneity, *T2’* T2 prime

When focusing on perfusion restricted areas with a TTP delay ≥ 2 s, median ROI volume was 16.9 mL (IQR, 13.6–30.5 mL). In these regions, median CBF (52.04 mL/100g per min, IQR 30.24–97.74 mL/100g per min versus 57.76 mL/100g per min, IQR 28.67–101.40 mL/100g per min, *p* = 0.001) was significantly decreased in the ipsilateral side to stenosis as compared to the corresponding contralateral regions at baseline. On the other hand, median CTH (3.55 s, IQR 2.78–7.49 s versus 3.14 s, IQR 2.52–6.42 s, *p* = 0.008), OEC (0.47, IQR 0.40–0.61 versus 0.44, IQR 0.40–0.56, *p* = 0.008) and MTT (3.25 s, IQR 2.48–6.35 s versus 2.80 s, IQR 2.33–5.25 s, *p* = 0.010) were significantly increased (see Table 3 and supplemental figure 2). The ICA revascularization led to a significant increase of 6.8% in nCBF (*p* = 0.017) accompanied by a significant reduction of 3.3% in nOEC (*p* = 0.033), 6.8% in nCTH (*p* = 0.048) and 6.4% in nMTT (*p* = 0.027). For the same perfusion restricted regions, nqT2’, nCBV, nCMRO_2_ and nRTH remained unchanged. The longitudinal comparison of non-normalized values did not show any significant differences (see Supplemental Table 2). The changes described above are displayed in Fig. 3 and Supplemental Table 3. The median white matter hyperintensities lesion load was 3.281 mL, IQR 9.234 mL (Fig. 4).

**Table 3:** Hemisphere comparison of non-normalized parameters in areas with TTP ≥ 2 s

	TP1 After 6–8 weeks			TP2		
	*Ipsi*	*Contra*	*p*	*Ipsi*	*Contra*	*p*
CBF _(mL/100 g per min)_	52.04 (67.50)	57.76 (72.73)	**0.001**	52.60 (82.56)	54.42 (84.76)	0.313
CBV _(mL/100g)_	116.03 (154.84)	123.86 (154.27)	0.216	123.09 (115.83)	114.96 (116.65)	1.000
MTT _(s)_	3.25 (3.87)	2.80 (2.92)	**0.010**	3.42 (4.65)	3.57 (4.61)	0.313
OEC _(%)_	0.47 (0.21)	0.44 (0.15)	**0.008**	0.48 (0.18)	0.48 (0.18)	0.461
CTH _(s)_	3.55 (4.71)	3.14 (3.9)	**0.008**	3.81 (7.16)	3.68 (6.92)	0.313
CMRO_2 (mL/100_ _mL per min)_	2.78 (2.96)	2.78 (3.04)	**0.057**	2.78 (2.92)	2.83 (2.93)	0.640
T2’ _(ms)_	116.33 (37.29)	116.34 (44.15)	0.380	114.20 (36.21)	113.21 (43.68)	0.109
RTH	1.10 (0.22)	1.10 (0.22)	0.541	1.14 (0.42)	1.14 (0.42)	0.313

Non-normalized values are presented as median (interquartile range). ‘ipsi’ indicates regions of interest in the hemisphere ipsilateral to the stenosis while ‘contra’ refers to the regions of interest in the hemisphere contralateral to the stenosis. Statistically significant *p*-values are presented in bold.

*CBF* cerebral blood flow, *CBV* cerebral blood volume, *MTT* median transit time, *OEC* oxygen extraction capacity, *CTH* capillary transit time heterogeneity, *CMRO*_*2*_ cerebral metabolic rate of oxygen, *T2’* T2 prime, *RTH* relative transit time heterogeneity

### Cognitive Testing

Mean cognitive performance scores are presented in Table 1. No significant differences were observed in patients before and after revascularization. Moreover, no statistically significant correlations were found between cognitive test results and hemodynamic metrics at baseline or follow-up after correcting for multiple testing.

## Discussion

In this study hemodynamic and oxygen metabolic profiling derived from DSC perfusion-weighted imaging along with qT2’ mapping were applied to investigate changes of cerebral oxygen metabolism and microvascular function before and 2–3 months after carotid revascularization in patients with ICAS. In line with a previous report [31], this analysis shows that ICA revascularization improves CBF and other perfusion metrics such as MTT, TTP and delay. In addition, carotid intervention resulted in sustained normalization of OEC and CTH 2 months after revascularization.

A moderate but compensated stage of hemodynamic impairment was observed at baseline. The CMRO_2_ is preserved in perfusion-restricted regions ipsilateral to stenosis owing to an increase in OEC, which in turn is the result of reduced CBF, elevated CTH, or both, in the affected carotid territories as compared to the contralateral side. In a recent study with a similar asymptomatic cohort, a reduction of CBF and CMRO_2_ was observed in the MCA territory ipsilateral to stenosis, whereas estimated relative OEF remained unchanged, leading to a disruption in the interhemispheric CMRO_2_-CBF coupling when compared to healthy controls [32]. Although not having conducted a local flow-metabolism analysis, these findings seem to suggest a maintenance of this coupling in perfusion-restricted areas despite high-grade stenosis, as CMRO_2_ was not altered owing to a compensatory increase in OEC. This inconsistency may rely on differences in the calculation of the hemodynamic parameters between both studies, as Göttler et al. measured absolute CBF by pseudocontinuous arterial spin labeling and relative OEF was calculated from R2’ and CBV (as opposed to the DSC-derived CBF and OEC in this study).

No significant changes were found between hemispheres at baseline or between timepoints in qT2’ values, contradicting previous studies using this technique in patients with mixed (i.e. symptomatic and asymptomatic) unilateral carotid stenosis [12, 13]. This can be explained by the selection of asymptomatic patients without ischemic brain lesions and overall less pronounced perfusion deficits. The lack of a motion correction technique applied to our R2 and R2* acquisitions could have also influenced our results. The T2’ signal variation results mainly from shifts in the capillary concentration of deoxygenated Hb, which in turn depends on the net balance between oxygen supply and consumption [33]. An increase in CBV can lead to an accumulation of deoxygenated Hb and theoretically induce a decrease in T2’ signal. In chronic hypoperfusion, cerebral autoregulation increases CBV in an attempt to compensate for decreased CBF. The T2’ signal is thus expected to be inversely proportional to both OEF and CBV. Interestingly, no statistically significant difference in baseline CBV between hemispheres was found, leading to the belief that OEC elevation was not marked enough to drive a significant change of qT2’ values and these may indeed be independent from CBV changes, as already reported in a previous study [34].

The microvascular distribution of blood was further addressed through measurement of CTH and relative CTH (RTH), i.e. the CTH:MTT ratio. The latter corresponds to the coefficient of variation of the intra-voxel transit time distribution [35] and can be seen as a measure of relative flow heterogeneity. Passive and compliant microvascular networks ensure a proportional change of CTH in relation to MTT [36], meaning that RTH remains constant. Gradual changes in the microvascular configuration lead to a functional shunting of blood through the capillary bed, which in turn ensures a wider distribution of transit times (increases CTH) and hampers oxygen extraction [15]. This becomes critical if CTH exceeds MTT, reflecting an increase of RTH along with CTH. The RTH is thus particularly useful in distinguishing passive blood vessel diameter changes due to effects of hypoperfusion from active CTH elevations in the context of structural and morphological microvascular impairment, which in turn leads to functional shunting and decrease in oxygen extraction efficacy.

In this cohort, while both CTH and MTT were increased in the pre-revascularization setting and subsequently normalized after carotid intervention, RTH remained unchanged. These findings are most likely the result of a compliant microvascular bed, where changes in blood distribution are passive and driven by the effects of hypoperfusion. The mismatch between CTH and MTT in the pre-interventional setting reflects the high variability of RTH observed and indicates some level of microvascular shunting through the capillary bed. The restoration of disturbed CTH after revascularization further suggests that the microvascular dysfunction is functional rather than structural. Besides justifying the compensatory increase of OEC, these findings go in line with the results of Arsava et al. in a sample of symptomatic and asymptomatic individuals [18]. In the latter study, a pre-interventional mismatch between CTH (increased) and RTH (decreased) was observed, with subsequent short-term normalization (within 24 h) after revascularization. The unbalance between microvascular and macrovascular hemodynamics was interpreted either as a compensatory homogenization of transit times to improve oxygen extraction or the presence of occluded but not structurally altered capillaries in hypoperfused tissue. This can also be the case in our patient cohort, thereby extending the hypothesis of a functionally altered microvascular compartment in hypoperfused tissue to asymptomatic patients with no evidence of prior ischemic lesions.

In another recent study with a cohort of asymptomatic ICAS, CTH increases were found to be diffuse in the white matter and independent of watershed areas [6], complementing our results by suggesting that impairments of capillary function go beyond areas with TTP ≥ 2 s.

Just like functional connectivity [37], neurocognitive function has been shown to be impaired in patients with asymptomatic carotid stenosis [9, 10]. Although lacking clinical significance, the status of compensated hemodynamic impairment and apparent microvascular dysfunction observed in our patients may suggest that such reversible microvascular changes precede the onset of cognitive impairment. This is mere speculative as this study was not statistically powered for cognitive evaluation and the tests used lack sensitivity.

To the best of our knowledge, this was the first study to investigate the early mid-term evolution of oxygen-metabolic and microvascular parameters after carotid revascularization in a cohort of exclusively asymptomatic unilateral carotid disease without evidence of ischemic brain lesions.

This study has several limitations. The small sample size and absence of a control group limits the statistical power and external quality of the study. The use of interhemispheric ratios should be interpreted with care, as they can be influenced by collateral status and longitudinal changes in the hemispheres contralateral to stenosis, thereby biasing the longitudinal analysis. Even though OEC and qT2’are supposed to be oxygen-sensitive, both parameters behaved differently across hemispheres and timepoints. These parameters have different calculation methods and, presumably, underlying physiological substrates. The estimation of OEF through T2’ imaging is based on CBV [38] and this calculation was not performed. Besides, the substantial partial volume artefacts in T2’ images and consequent need of thresholding also presents a limitation to the analysis of these images. The interpretation of both T2’ and DSC-based OEC is still largely unclear and both methods require further investigation and validation with ^15^O‑PET, the gold standard for imaging cerebral oxygen metabolism. Another limitation of this study is the fact that DSC provides an estimation of CBF (as opposed to a quantitative CBF calculated by arterial spin labeling), hence influencing CMRO_2_ values.

## Conclusion

In this study, a state of altered microvascular function was identified in patients with high-grade ICAS in the absence of ischemic brain lesions. Internal carotid artery revascularization led to sustained normalization of cerebral hemodynamics and restoration of capillary function. Further studies should address capillary homeostasis in larger cohorts and at longer intervals after revascularization with the aim to optimize personalized stroke risk assessment and patient selection for therapy.

## Supplementary Information

Supplemental Table 1: Longitudinal comparison of non-normalized parameters in the middle cerebral artery (MCA) territory; Supplemental Table 2: Longitudinal comparison of non-normalized parameters in areas with time-to-peak (TTP) delay ≥ 2 s; Supplemental Table 3: Longitudinal comparison of normalized parameters

**Supplemental Fig. 1** Illustration of hemodynamic and metabolic maps from two subjects with ICAS (internal carotid artery stenosis) before and after carotid revascularization.

**Supplemental Fig. 2** Boxplots for non-normalized hemodynamic and oxygen-metabolic parameters (CBF, CBV, CMRO_2_, OEC, CTH, qT2’, MTT, RTH) values obtained from areas with a TTP delay ≥ 2 s of the side ipsilateral to stenosis (ipsi) as well as contralateral (contra) side. For better visualization, outliers are not included in the plots, but are naturally included in the statistical analysis. The Y‑axes represent the absolute values of each parameter.
