# Supplementary material for: Comprehensive Evaluation of Cerebral Hemodynamics and Oxygen Metabolism in Revascularization of Asymptomatic High-Grade Carotid Stenosis
Source: Clin Neuroradiol. 2021 Sep 6;32(1):163–73. doi: 10.1007/s00062-021-01077-3 (PMC8894147; doi:10.1007/s00062-021-01077-3)
Supplement: Supplementary file 1 — Supplemental Table 1: Longitudinal comparison of non-normalized parameters in the middle cerebral artery (MCA) territory; Supplemental Table 2: Longitudinal comparison of non-normalized parameters in areas with time-to-peak (TTP) delay ≥ 2 s; Supplemental Table 3: Longitudinal comparison of normalized parameters [file 62_2021_1077_MOESM1_ESM.docx]

# Supplemental Table 1 – Longitudinal comparison of non-normalized parameters in the MCA territory:

|  | **ipsi**  **After 6-8 weeks** | | | **contra** | | |
| --- | --- | --- | --- | --- | --- | --- |
|  | *TP1* | *TP2* | *p* | *TP1* | *TP2* | *p* |
| CBF _(mL/100g/min)_ | 97.28 (64.05) | 76.29 (82.47) | 0.893 | 97.38 (97.70) | 75.93 (85.62) | 0.839 |
| CBV _(mL/100g)_ | 177.42 (228.29) | 164.54 (133.12) | 0.339 | 173.71 (224.22) | 162.29 (137.58) | 0.339 |
| MTT _(s)_ | 3.05 (3.19) | 3.45 (3.67) | 1.000 | 2.76 (2.52) | 3.51 (3.65) | 0.376 |
| OEC _(%)_ | 44.59 (18.23) | 46.94 (15.87) | 1.000 | 42.46 (14.61) | 47.59 (16.74) | 0.305 |
| CTH | 3.41 (4.37) | 3.85 (5.89) | 0.839 | 3.05 (3.91) | 3.87 (5.65) | 0.541 |
| CMRO_2 (mL/100mL/min)_ | 4.25 (3.26) | 3.97 (3.01) | 0.414 | 4.24 (3.15) | 4.04 (3.20) | 0.588 |
| T2’ _(ms)_ | 112.78 (25.92) | 109.35 (20.67) | 0.129 | 111.84 (30.22) | 109.85 (26.46) | 0.109 |
| RTH | 1.13 (0.26) | 1.16 (0.45) | 0.376 | 1.13 (0.30) | 1.18 (0.42) | 0.893 |

Non-normalized values obtained before (TP1) and 6-8 weeks after carotid revascularization (TP2) are presented as median (IQR). ‘ipsi’ indicates regions of interest in the hemisphere ipsilateral to the stenosis while ﻿‘contra’ refers to the regions of interest in the hemisphere contralateral to the stenosis.

**Supplemental Table 2 – Longitudinal comparison of non-normalized parameters in areas with TTP ≥ 2s:**

|  | **ipsi**  **After 6-8 weeks** | | | **contra** | | |
| --- | --- | --- | --- | --- | --- | --- |
|  | *TP1* | *TP2* | *p* | *TP1* | *TP2* | *p* |
| CBF _(mL/100g/min)_ | 52.04 (67.50) | 52.60 (82.56) | 0.110 | 57.76 (72.73) | 54.42 (84.76) | 0.635 |
| CBV _(mL/100g)_ | 116.03 (154.84) | 123.09 (115.83) | 0.893 | 123.86 (154.27) | 114.96 (116.65) | 0.588 |
| MTT _(s)_ | 3.25 (3.87) | 3.42 (4.65) | 0.735 | 2.80 (2.92) | 3.57 (4.61) | 0.414 |
| OEC _(%)_ | 47.11 (21.17) | 47.51 (18.28) | 0.787 | 43.62 (15.83) | 47.55 (18.68) | 0.497 |
| CTH | 3.55 (4.71) | 3.81 (7.16) | 0.787 | 3.14 (3.9) | 3.68 (6.92) | 0.376 |
| CMRO_2 (mL/100mL/min)_ | 2.78 (2.96) | 2.78 (2.92) | 0.094 | 2.78 (3.04) | 2.83 (2.93) | 0.191 |
| T2’ _(ms)_ | 116.33 (37.29) | 114.20 (36.21) | 0.622 | 116.34 (44.15) | 113.21 (43.68) | **0.042** |
| RTH | 1.10 (0.22) | 1.14 (0.42) | 0.340 | 1.10 (0.22) | 1.14 (0.42) | 0.497 |

Non-normalized values obtained before (TP1) and 6-8 weeks after carotid revascularization (TP2) are presented as median (IQR). ‘ipsi’ indicates regions of interest in the hemisphere ipsilateral to the stenosis while ﻿‘contra’ refers to the regions of interest in the hemisphere contralateral to the stenosis.

# Supplemental Table 3 – Longitudinal comparison of normalized parameters

|  | **MCA territory** | | | **TTP ≥2s** | | |
| --- | --- | --- | --- | --- | --- | --- |
|  | *TP1* | *TP2* | *p* | *TP1* | *TP2* | *p* |
| CBF | 0.990 ± 0.043 | 1.027 ± 0.020 | 0.190 | 0.906 ± 0.088 | 0.967 ± 0.050 | **0.017** |
| CBV | 1.024 ± 0.040 | 1.018 ± 0.049 | 0.569 | 0.954 ± 0.096 | 0.989 ± 0.051 | 0.244 |
| MTT | 1.081 ± 0.093 | 1.019 ± 0.049 | **0.007** | 1.088 ± 0.104 | 1.036 ± 0.039 | **0.027** |
| OEC | 1.046 ± 0.050 | 1.001 ± 0.022 | **0.007** | 1.037 ± 0.050 | 1.017 ± 0.022 | **0.033** |
| CTH | 1.071 ± 0.074 | 1.024 ± 0.049 | 0.065 | 1.091 ± 0.092 | 1.025 ± 0.039 | **0.048** |
| CMRO_2_ | 1.012 ± 0.028 | 1.009 ± 0.029 | 0.380 | 0.936 ± 0.078 | 0.975 ± 0.053 | 0.244 |
| T2’ | 1.003 ± 0.046 | 1.009 ± 0.050 | 0.642 | 1.001 ± 0.042 | 1.012 ± 0.062 | 0.233 |
| RTH | 1.000 ± 0.020 | 1.003 ± 0.010 | 0.463 | 0.999 ± 0.010 | 1.004 ± 0.007 | 0.497 |

Normalized values obtained before (TP1) and 6-8 weeks after carotid revascularization (TP2) values are presented as mean±SD.
