# Supplementary figures and images for: Comprehensive Evaluation of Cerebral Hemodynamics and Oxygen Metabolism in Revascularization of Asymptomatic High-Grade Carotid Stenosis
Source: Clin Neuroradiol. 2021 Sep 6;32(1):163–73. doi: 10.1007/s00062-021-01077-3 (PMC8894147; doi:10.1007/s00062-021-01077-3)

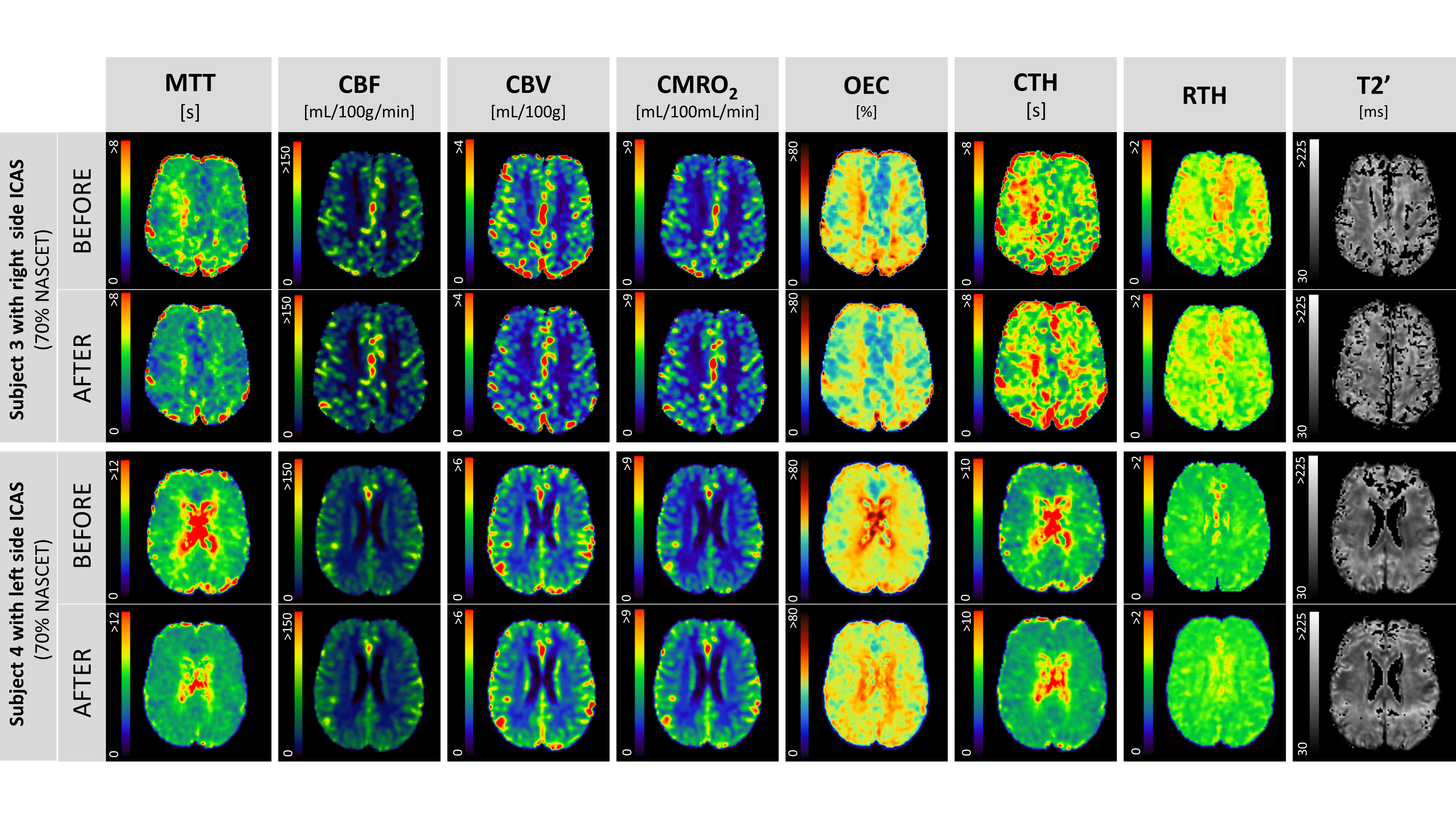

Supplement: Supplementary file 2 — Supplemental Fig. 1 Illustration of hemodynamic and metabolic maps from two subjects with ICAS (internal carotid artery stenosis) before and after carotid revascularization. [file 62_2021_1077_MOESM2_ESM.tif]
